# Novel strategies for immuno-oncology breakthroughs with cell therapy

**DOI:** 10.1186/s40364-021-00316-6

**Published:** 2021-07-31

**Authors:** Hongtao Liu, Chongxian Pan, Wenru Song, Delong Liu, Zihai Li, Lei Zheng

**Affiliations:** 1 Chinese American Hematologist and Oncologist Network, New York, NY USA; 2 University of Chicago, Chicago, IL USA; 3 Harvard University, Boston, MA USA; 4 Kira Pharmaceuticals, Cambridge, MA USA; 5 New York Medical College, Valhalla, NY USA; 6 Pelotonia Institute for Immuno-Oncology, The Ohio State University, Columbus, OH USA; 7 Johns Hopkins University, Baltimore, MD USA

**Keywords:** Cellular Therapy, CAR-T, TCR-T, Neoantigen, TME

## Abstract

Cell therapy has evolved rapidly in the past several years with more than 250 clinical trials ongoing around the world. While more indications of cellular therapy with chimeric antigen receptor – engineered T cells (CAR-T) are approved for hematologic malignancies, new concepts and strategies of cellular therapy for solid tumors are emerging and are discussed. These developments include better selections of targets by shifting from tumor-associated antigens to personalized tumor-specific neoantigens, an enhancement of T cell trafficking by breaking the stromal barriers, and a rejuvenation of exhausted T cells by targeting immunosuppressive mechanisms in the tumor microenvironment (TME). Despite significant remaining challenges, we believe that cell therapy will once again lead and revolutionize cancer immunotherapy before long because of the maturation of technologies in T cell engineering, target selection and T cell delivery. This review highlighted the recent progresses reported at the 2020 China Immuno-Oncology Workshop co-organized by the Chinese American Hematologist and Oncologist Network (CAHON), the China National Medical Product Administration (NMPA), and Tsinghua University.

## Introduction

Cancer immunotherapy has been a game changer in cancer treatment since the approval of the immune checkpoint inhibitor (ICI), ipilimumab, in 2011 and subsequent anti-PD-1 antibody, pembrolizumab and nivolumab in 2014 [1–5]. Currently, 10 immune checkpoint inhibitors and 5 chimeric antigen receptor T cell (CAR-T) products have been approved in treating 17 types of malignant diseases and two tissue-agnostic indications across the world [6–12]. CD19-targeted CAR-T cell therapy for B cell neoplasms has opened up a new era in synthetic cancer immunotherapy [13–15]. At the time of the workshop, there are three approved CD19-CAR-T cell platforms: tisagenlecleucel (Kymriah) for B cell lymphoma and pediatric B cell acute lymphocytic leukemia (ALL), axicabtagene ciloleucel (Yescarta) for B cell lymphoma, and brexucabtagene autoleucel (Tecartus) for mantle cell lymphoma (MCL) [16–18]. Two new CAR-T cell products have since received the FDA approval [19, 20]. Besides CD3ζ chain, Tisagenlecleucel uses CD137 (4-1BB) as an additional co-stimulating signal (COS), while axicabtagene ciloleucel and brexucabtagene autoleucel use CD28 as second COS [21]. All agents utilize a single chain anti-CD19 fragment to target malignant B cells. Tisagenlecleucel is approved for the treatment of patients up to 25 years of age with B cell precursor ALL that is refractory to standard therapy or in at least second relapse. Both tisagenlecleucel and axicabtagene ciloleucel are indicated for the treatment of relapsed or refractory large B cell lymphoma. Brexucabtagene autoleucel (Tecartus) is indicated for adult patients diagnosed with MCL. Other development platforms of cellular therapies include tumor infiltrating lymphocytes (TIL), natural killer (NK) cells, CAR-NK cells, T cells expressing T cell receptor (TCR) ectopically (TCR-T), which are expanding the cell therapy indications from the hematologic malignancies to solid tumors [22–25]. Cellular therapy has been an important aspect of every immuno-oncology conference including the past five annual China Cancer Immunotherapy Workshops [5]. In 2020, the China Cancer Immunotherapy Workshop provided comprehensive updates on the current progresses and future prospects in cellular therapy for malignant diseases.

### CAR-T cell therapy for hematologic malignancies: the legacy continues

The clinical development of CAR-T cell therapy for hematologic malignancies was exampled by the recent approval of CD19-targeting KTE-X19, brexucabtagene autoleucel, for a new indication, the mantle cell lymphoma (MCL). The treatment for MCL has evolved in past decades from chemotherapy to chemotherapy-free targeted therapies and now to cellular therapies with the latest approval of brexucabtagene autoleucel [26, 27]. The Phase II ZUMA-2 multi-center, international study enrolled 74 patients with relapsed and refractory MCL [18]. Among these 74 patients who underwent leukopheresis, 69 patients received conditioning chemotherapy; and 68 patients received KTE-X19. Thus, KTE-X19 was successfully manufactured for 96 % of patients with a median time of 16 days from leukopheresis to delivery of products to the study site. These patients were heavily pretreated with a median number of prior therapies being three. All these patients failed anti-CD20 antibody and BTK inhibitor treatments in the past. The study demonstrated an ORR of 93 % and a CR of 67 %; and the response was consistent across all the key subgroups. The response was also durable; the median duration of response was not reached after a median follow-up of 12.3 months. As many as 57 % of all patients and 78 % of patients who achieved CR remained in remission at the last follow-up. The median PFS and OS were not reached after a median follow-up of 12.3 months. The safety profile in this study is consistent with that reported in prior studies of anti-CD19 CAR-T cell therapies in NHL. There were no deaths due to cytokine release syndromes (CRS) or neurologic events. Most of side effects occurred during the early course of treatment and were generally reversible. The degree of CAR-T cell expansion *in vivo* correlated with clinical efficacy and toxicities.

Beyond the currently approved CAR-T cells targeting CD19 for lymphocytic leukemia and lymphomas, at this workshop, Marcela Maus discussed several new targets. One of them, CD37, is a tetraspanin family protein (TSPAN26) which functions in cell membrane organization and co-signaling, modulating cellular adhesion, mobility and proliferation, and providing both pro-survival and pro-apoptotic signals. CD37 is expressed on multiple tumor subtypes, including B cell NHL, CLL, Waldenstrom’s macroglobulinemia, T-ALL, PTCL (reported 82 % positive for CD37), and AML [28–30]. High CD37 expression was also confirmed in patient derived xenografts (PDX) of mantle cell lymphoma, CLL, and PTCL by flow cytometry and immunohistochemistry. The CD37 CAR-T cells were as effective as CAR19 in Jeko and PDX model of mantle cell lymphoma [31]. Based on these promising preclinical data, an early phase clinical trial was opened to explore the CD37 CAR-T cells for CD37^+^ hematologic malignancies, including mature B cell neoplasms, mature T cell neoplasms, and T-cell or B-cell prolymphocytic leukemia (NCT04136275).

For multiple myeloma (MM), the anti-BCMA (B cell maturation antigen) CARs, which are under extensive clinical development [32–34], have two issues: they could be rejected due to the presence of non-human sequences; there could be emergence of variants from loss of BCMA expression on the MM cells. Thus, better and cancer-specific target antigens are still desired for CAR-T. Soluble APRIL (a proliferation inducing ligand), which is produced by myeloid cells in the bone marrow microenvironment could bind both BCMA and TACI (transmembrane activator and CAML interactor) to promotes survival and differentiation of plasma cells and MM cells. Thus, APRIL could be used to guide CAR-T cells to target both BCMA and TACI [35]. CAR-T cells expressing monomeric APRIL had limited clinical efficacy, but those expressing trimeric APRIL not only were able to eliminate BCMA^+^ MM, but also able to eliminate BCMA^-^ MM in the animal models [36]. Thus, a clinical trial using the CAR-T cells expressing trimeric APRIL is being planned for MM patients regardless of BCMA expression.

There has been an explosive growth of CAR-T clinical trials, currently with 245 trials in China and 167 trials in the US. Current development of CAR-T therapy for hematologic malignancies at one aspect is focusing on modifying CARs structurally, including the addition of multiple antigen binding capacities with mono-antibodies, humanized mono-antibody or nano-body with different affinities, testing different hinges to connect the antibody with the transmembrane domain, optimizing different co-stimulatory domains (4-1BB, CD28, GITR, CD27), and incorporating mutations in the signaling domains to tune the signaling intensity. Second, different immune effector cells are tested to replace the traditional T cells including γδ T cells, NK cells, and off-the-shelf CAR-T cells based on gene editing. In addition, different gene delivery technologies are available for engineering CAR-T cells [37, 38]. Third, further modification of the CAR-T cells are made with additional transgenes to express cytokines to stimulate CAR-T cell function and maintain their persistence, with gene editing to knock out checkpoint molecules, and with controlled expression of CARs through a “switch” on and off mechanism [39]. Fourth, the strategies to engineer CAR-T cells for improved safety and minimize resistance [40–42]. These strategies include combinatorial antigen recognition by a synthetic Notch receptor on CAR-T cell to increase antigen specificity and thus safety and engineered ON and OFF switches that can promptly and efficiently alter CAR-T cell activity by programming CARs to be activated only in the presence of an adaptor or by leucine-zipper-mediated reconstitution [40]. The latter is achieved by co-expression of suicide genes such as HSK-TV, iCasp9, CD20, and tEGFR that enable induction of T-cell death to abort the therapy in case of severe adverse events with a Tet-ON and -OFF system to allow the control of the CAR expression on the transcriptional level. These strategies are summarized in Table 1.

**Table 1:** Strategies to improve the efficacy and mitigate the toxicities in cellular therapies

Area	Challenges	Potential solutions
**Platform**	Failure to manufacture of engineered T cells	Allogeneic or Universal CAR-T or TCR-T with gene editing [43]
	Lack of efficacy of CAR-T in solid tumors	TIL, TCR-T, NK CAR-T [44]
	Lack of persistence of T cells	Self-secretion of cytokines to maintain survival of proliferation of engineered T cell, like IL-2, IL-15 et al. [40]
	Lack of efficacy	Third generation with dual co-stimulatory signals [45] PD-1 knockout, or expression of PD-1 DN [46] Selection of specific T cell population, like γδ T cells, CD8^+^CD39^−^CD69^−^ T cells [47].
**Target**	Lack of target	Tumor neoantigens, individualized cell therapy [48]
	Loss of target	Dual targets CAR-T [49] Sequential administration of CAR-T targeting different antigen [40]
	Antigens shared by tumor and normal cells, like hematopoietic stem cells	Low affinity CAR-T, TCR-T to avoid killing normal cells with low expression level [50] Cellular therapy followed by stem cell transplant using gene-knockout, like CD33^-^ hematopoietic stem cells [51]
**T cell trafficking**	Lack of trafficking of T cells to tumor site	Intra-tumor or intra tumor site (intra-pleural) administration of cellular therapy [52] Chemotherapy (like oxaliplatin and cyclophosphamide) and/or local tumor radiation prior to infusion of T cells [53]. Expression of chemokines by the engineered T cells [40]
**T cell functions**	T cell exhaustion at the TME	PD-1 knockout or expression of PD-1 DN [46] Re-direction of Treg by BiTEs [54] Administration of checkpoint inhibitor after T cell infusion [55] STING agonists [56] Small molecules and mAbs targeting the CSF-1/CSF-1R axis to decrease suppressive macrophages [57]
**Mitigation of toxicities**	CRS and ICANS	Combinatorial antigen recognition by AND and AND-NOT logic using a synNotch receptor and iCAR [40]. Off-switch receptors or inducible suicide constructs [40]

Moreover, one recent focus of the clinical development was to improve the manufacturing of CAR-T cells. With a new platform designated FasT, CAR-T cell manufacture time is shortened to one day. The early phase study had demonstrated that CAR-T cells on the FasT platform has a superior expansion capability and less exhausted phenotypes, and were safe and highly effective for treating patients with B-ALL [58].

### From tumor infiltrating lymphocyte therapy to individualized TCR-T Cell therapy

Steven Rosenberg devoted his research to developing more effective immunotherapies for cancer based on the adoptive transfer of immune cells with anti-cancer activity [59]. The advantages of cell transfer therapy include being able to (1) administer large numbers of highly selected cells with high avidity for tumor antigens, (2) administer cells activated *ex-vivo* to exhibit anti-tumor effector function *in vivo*, (3) selecting specific immune cell subpopulations with anti-cancer effector function, and (4) manipulate the host prior to cell transfer to provide altered immune system and favorable tumor micro-environment (TME) for transferred cells.

The concept of adoptive cell transfer immunotherapy was initially tested in solid tumors, but it made the first impact on the treatment of hematologic malignancies. The first lymphoma patient was treated with autologous anti-CD19 CAR-T cells at the NCI in 2009 [60, 61]. The patient had failed multiple lines of treatment before he received anti-CD19 CAR-T cells; and since then, the patient has remained progression-free for 10 years. Among this cohort of patients with refractory diffuse large B-cell lymphoma, 73 % patients had an objective response including complete response (CR) of 47 % and durable CR of 42 %.

Compared to hematologic malignancies, solid cancers cause higher death numbers every year. The major challenge thus confronting cancer immunotherapy is to develop effective immunotherapies for patients with metastatic epithelial solid cancers that cannot be cured by any available treatments. Since CAR-T cells require the use of monoclonal antibodies (MoAb) that recognize molecules on the cell surface, there are two major challenges for CAR-T cells for the treatment of solid epithelial cancers: (1) very few MoAbs exist that recognize cell surface molecules unique to cancer (e.g., EGFRvIII); (2) normal cells expressing the target of MoAb are highly sensitive to destruction. Most MoAbs in clinical use target growth factors, such as EGFR or Her-2 that are expressed on the surface of normal cells.

Steven Rosenberg reported many unpublished data at this 2020 China Cancer Immunotherapy Workshop, including treatment of 194 patients with metastatic melanoma using tumor infiltrating lymphocytes (TIL). Among these patients, 63 patients (31 %) had partial response (PR), and 46 patients (24 %) had CR, with ORR of 55 %. 44 of 46 CR patients have remained CR for 14 to 152 months following a single treatment. With a median follow of 6.3 years, the estimated 10-year OS of all 194 patients is approximately 35 %; and the estimated 10-year OS of the 46 patients who achieved CR is > 90 %, suggesting that adoptive cell transfer immunotherapy is able to provide a cure or a long-term survival to metastatic melanoma patients.

More recently, the Rosenberg group started to explore the cancer neoantigens as the targets of the TIL therapy. In summary, for a mutation to be a cancer antigen for a CD8^+^ T cell, it has to be processed intracellularly into a 9–11 amino acid peptide; and such a peptide must be presented to the MHC class I molecules to bind to the T cell receptor (TCR) on CD8^+^ T cells. Thus, not all the mutated peptides will be antigenic. The Rosenberg group has developed a T cell expansion protocol for the generation of mutation-reactive T cells for the common malignant diseases. The protocol starts with the whole exome and transcriptome sequencing of tumor cells to identify mutations, followed by synthesis of mutated tandem minigenes (TMGs) that each encodes a 25 amino acid peptide and together cover all the mutations. TMGs are transfected into autologous APC (e.g., dendritic cells). In parallel, TILs are isolated from patients and expanded *ex vivo* in presence of IL-2. Then, APCs loaded with each TMG will be co-cultured with TILs and, by using INF-γ production and 4-1BB/OX40 upregulation as readouts, to identify the mutations which induce T cell activation [62, 63]. There are a few advantages of this approach, including that there is no need to predict the peptide binding to MHC as all candidate peptides and all MHC loci are included in the screening assay and there is no need to use patient-derived tumor cell lines. This study evaluated 22 patients with melanoma, and the team identified a total of 13,664 mutations with a median of 318 mutations in each patient. 54 immunogenic neoepitopes were identified after screening of 3,938 mutations, suggesting that the chance for a mutation being an immunogenic neoepitope could be as low as 1.4 % (54/3,938). The majority of the 54 neoepitopes are CD8^+^ T cell epitopes (94 %); and 6 % are CD4^+^ epitopes. Among 22 patients evaluated, 18 (82 %) demonstrated mutation-reactive T cells in their TILs. Every neoantigen identified was unique to individual patient; and none was shared by the patients. In addition, 210 immunogenic mutations were identified by screening 15,256 mutations from 130 patients with gastrointestinal cancers. Among them, 47 % were reactive to CD8^+^ T cells; and 53 % reactive to CD4^+^ T cells. Every neoantigen was unique to individual patients except 2 patients who shared the same KRAS mutation-specific neoantigen. Moreover, the Rosenberg group screened 195 patients with other types of cancers and identified a total of 363 neoantigens; and 77 % of these 195 patients demonstrated the neoantigen reactivity in the TILs. Among these 363 neoantigens, each was unique to individual patients except for two KRAS mutation specific neoantigens. These results suggest that common oncogenic driver mutations such as mutations in KRAS, TP53, etc. can be shared neoantigens.

Successful treatments of individual patients with the above TIL therapy were reported. One patient is a 45 year old female who has a cholangiocarcinoma with multiple lung and liver metastases and failed two lines of chemotherapy and an attempted treatment with unselected TILs. Twenty-six neoantigens were identifies through the TMGs approach in her tumor. Her TILs were ex vivo enriched with ERBB2IP mutation-reactive CD4^+^ T lymphocytes and generated an ongoing objective response in lung and liver metastases over 7 years [63]. Another one is a 51 year old female with metastatic breast cancer who failed 7 lines of treatments prior to the TIL therapy. A total of 62 mutations were identified in her tumor. She has an ongoing CR over 58 months after the treatment with 8 × 10^9^ TILs plus 7 doses of IL-2 and 4 doses of anti-PD-1 antibody, Pembrolizumab [64]. About 23 % of her TILs were found to be reactive to four mutations, including SL3A2, KIA0368, CADPS2, and CTSB. The neoantigen-specific TCRs were still present in her body at 6 weeks after the initial infusion of TILs. One patient with metastatic cervical cancer [48] and another patient with metastatic colon cancer also had an excellent response. In summary, among patients with chemotherapy refractory metastatic solid tumors, 0/20 patients had response to the TILs treatment, 3/25 (12 %) had response to neoantigen-selected TILs, and 6/26 (23 %) had response with neoantigen-selected TILs plus an anti-PD-1 antibody, pembrolizumab. These results are promising; however, there are still challenges to overcome in order to make the TIL therapy available for the majority of cancer patients. It was speculated that the randomly occurred somatic mutations are likely the “common pathway” that underlies the cancer regression from most of immunotherapies for solid cancers, including IL-2, anti-CTLA4 antibody, anti-PD-1 antibody, anti-CD40 antibody and TIL therapies. In theory, virtually all cancer patients are potentially eligible for TIL-based therapies. The challenge is that the TIL therapy will have to be highly individualized and thus is resource-heavy.

The Rosenberg group characterized the TILs that mediate cancer regression *in vivo* using high dimensional single cell transcriptomic tSNE (t-distributed stochastic neighbor embedding) analysis. The CD45^+^CD3^+^ population of TILs were clustered into 22 subpopulations. Among them, only one cluster of cells could distinguish the responders from the non-responders; and this cluster of cells is significantly more abundant in the responders. Furthermore, this cluster of cells was highly enriched with CD39^-^CD69^-^ cells with a memory-progenitor feature, and were associated with better TIL persistence and clinical activity. By comparison, the terminally differentiated CD39^+^CD69^+^ T cells were associated with poorer TIL persistence.

Most of neoantigen-reactive TILs were found in the differentiated CD39^+^ status. However, TIL therapy responders retained a pool of CD39^-^ stem-like neoantigen-specific TILs that was lacking in TIL therapy non-responders. In the murine tumor TIL therapy model, CD8^+^CD39^-^CD69^-^ T cells could significantly suppress the tumor growth and prolong the survival of the mice to provide potential cure comparing to CD8^+^CD39^+^CD69^+^ T cells. Tumor-reactive stem-like TILs were capable of self-renewal, expansion, persistence, and provided superior antitumor response *in vivo* [47]. Several other strategies were also discussed to improve TIL efficacy, including but not limited to: increasing the frequency of neoantigen-reactive cells in the TIL infusion product by stably expressing antigen-specific TCRs in central memory T cells to generate TCR-T cells; combination therapy of TIL with anti-PD-1 antibody or other immune checkpoint inhibitors; and deleting PD-1 or other inhibitory molecules form the transferred T cells *via* gene editing.

In summary, two potentially most effective strategies for T cell therapy are enlightened by above TIL therapy studies. One is to target the immunogenic somatic mutations unique to individual patient’s cancer, ideally using T cells with memory-progenitor CD39^-^ stem-like phenotype; second is to generate a library of TCRs against shared cancer mutations from common oncogenes such as those in KRAS and TP53. Such strategies are applicable to the rapidly evolving development of TCR-T cell therapies that are anticipated to eventually replace TIL therapies [22, 23].

### Making CARs more accessible to solid tumors

During the 2020 China Cancer Immunotherapy Workshop, Prasad Adusumilli further described the current landscape of T cell therapies for solid tumors. At the time of the workshop, there were 84 active TCR-T cell therapy clinical trials, with the majority in melanoma and other solid tumors, only 5 % of clinical trials for patients with hematologic malignances. Among those clinical trials for solid tumors, all are in early phase except melanoma, four target cell surface molecules as shared antigens, and five target neoantigens [44, 65]. One of the approaches of cellular therapy for solid tumors is CAR-T cell therapy. Four strategies to achieve the success of CAR-T cell therapy in solid tumors are being explored: (1) selecting highly disease specific target antigens; (2) overcoming the barrier in the tumor immune microenvironment (TME) for a better delivery of CAR-T cells; (3) overcoming T cell exhaustion; (4) improving clinical trial designs [55]. The discussed CAR-T approaches for solid tumors during this conference were summarized in Table 2.

**Table 2:** Summary of the discussed CAR-T for solid tumors

Presenter	Target	Co-stimulatory molecule	Tumor type	Registration Number
Prasad Adusumilli	Mesothelin	CD28	Mesothelioma TNBC	NCT02414269
	Mesothelin	CD28 with PD-1 dominant negative receptor	Mesothelioma	NCT04577326
Marcela Maus	EGFRvIII	4-1BB	Glioblastoma	NCT02209376
Stanley Riddell	ROR1	4-1BB	ROR1 positive: Lung cancer TNBC Hematologic malignancies	NCT02706392

In this regard, multiple groups have demonstrated that mesothelin could be a disease-specific target antigen of CAR-T cell therapy for solid tumors. Mesothelin, as a cancer-associated cell surface antigen, is expressed in tumors of 20–85 % of patients of a variety of solid tumors [66]. Mesothelin expression is associated with tumor aggressiveness in several solid tumors, including mesothelioma, lung adenocarcinoma, triple negative breast cancer and esophageal cancer [67]. The MSKCC group has developed a fully humanized mesothelin-targeted CAR using either CD28 or 4-1BB as co-stimulation signals. Their scFv (single chain variable fragment) combining an IgG derived Fc and IgM derived Fv had a low affinity to bind mesothelin so as to spare low-mesothelin expressing normal tissues.

Many engineering strategies have been explored in CARs to overcome the barriers in the TME, including armoring CAR-T to enhance the infiltration of CAR-T cells into the tumors and genetic deletion of immune checkpoint molecules by gene editing tools, such as CRISPR/Cas9, from the CAR-T cells to avoid exhaustion [43, 45]. To study the TME, the Prasad Adusumilli group in collaboration with many others generated 2.5 million data points from 5,500 patients with 800 variables per patient over ten years. Through the analysis of infiltrating immune cells and chemokines, including type, number and location (tumor/stroma), they identified that the ratios of immune effectors to inhibitors are prognostic for the survival of patients, including the stroma CD3/FoxP3 ratio for lung adenocarcinoma [68], the tumor CD163/CD8 and CD163/CD20 ratios for mesothelioma [69], and the tumor CD10/CD20 ratio for lung squamous cell carcinoma [70]. Ongoing studies will expand the portfolios to include samples from the primary tumor sites, normal tissue, normal and metastatic lymph nodes, and blood samples. Other considerations include the heterogeneity of the tumor antigen expression and the distribution of CAR-T cells at the primary and metastatic sites which would both under the influence of TME and thus may impact the outcome of CAR-T cell therapy. In a preclinical study with the animal model, regional administration of CAR-T cell into pleura demonstrated significantly better disease control and survival than intravenous administration of CAR-T cells. Moreover, this study showed that regionally administrated CAR-T cells not only controlled the local disease, but also promoted efficient elimination of extrathoracic tumor sites. This therapeutic efficacy was dependent on early CD4^+^ T cell activation associated with a higher intratumoral CD4/CD8 cell ratio and CD28-dependent CD4^+^ T cell-mediated cytotoxicity. Interestingly, intravenously delivered CAR-T cells, even when accumulated at equivalent numbers in the pleural tumor, did not achieve comparable activation, tumor eradication, or persistence [52]. These results support the concept of delivering optimal CAR-T cell therapy through the regional administration. On the basis of these results, a phase 1 clinical trial to evaluate the safety of intrapleural administration of mesothelin-targeted CAR-T cells in patients with primary or secondary pleural malignancies has begun.

Mesothelioma has very low tumor mutational burden (TMB) and low PD-L1 expression which make it less responsive to the checkpoint inhibitor treatment [71] although checkpoint blockade is included in NCCN guideline as a second line of therapy due to limited treatment options for mesothelioma patients. The Prasad Adusumilli group leveraged their CAR-T cell therapy research by combining anti-PD-1 checkpoint blockade with CAR-T cell therapy to rescue the CAR-T cells from exhaustion, thus to enhance the CAR-T cell efficacy, and subsequently demonstrated that the combination improved the survival in the murine model [72]. Using the fully humanized mesothelin CAR with reduced immunogenicity, iCasM28z CAR, Adusumilli and his colleagues conducted two phase I clinical trials with intrapleural administration (NCT02414269) for malignant pleural mesothelioma (MPM) and with systemic administration (NCT02792114) for triple negative breast cancer, respectively. A total of 41 patients (4 patients received repeated doses) were treated intrapleurally and 10 patients (one patient received repeated doses) were treated systematically. There was no issue with CAR-T cell production, no Grade 3 or above adverse events, and no on-target off-tumor toxicities. A follow-up Phase II trial with intrapleural administration is ongoing. The phase 1 data were presented at 2019 AACR [73] and ASCO meeting [74]. A subset of mesothelioma patients received subsequent anti-PD-1 therapy, off-protocol, with an attempt to prolong CAR-T-cell functional persistence as previously suggested in the preclinical model [72]. At the data cut-off date (Jan 31, 2019), among 14 MPM patients who received both CAR-T cells and PD-1 blockade, 2 patients demonstrated complete metabolic response on PET scans; 5 demonstrated partial responses; and 4 demonstrated stable disease, all by investigator assessment. In one patient who had partial response after intrapleural administration of CAR-T cells, addition of anti-PD-1 antibody resulted in complete response that has lasted for 22 months and remains ongoing. In this patient, clonal expansion of CAR-T cells and non-CAR-T cells and antibody responses against new epitopes were observed both after CAR-T-cell infusion and after anti-PD1 therapy in several patients.

More recently, the next generation of CAR, designated M28z1XXPD1DNR CAR, expressing PD-1 dominant negative receptor on the surface of CAR-T cells to overcome the suppression by PD-L1 on the tumors, was developed. This new generation of CAR-T cells demonstrated enhanced *in vitro* cytotoxicity, and *in vivo* tumor killings to prolong survival in the animal model [72]. M28z1XXPD1DNR CAR-T cells had higher potency with lower dose of CAR-T cells and longer functional persistence *in vivo* than the traditional CAR-T cells. The clinical trial using this next generation CAR was initiated in September 2020 for patients with mesothelioma (NCT04577326).

For solid tumor CAR-T cell therapy, Marcela Maus provided another leading example of the current development. Her group conducted a phase I study of a single intravenous dose of EGFRvIII CAR-T cells in patients with recurrent EGFRvIII positive glioblastoma (GBM) (NCT02209376). EGFRvIII is an oncogenic mutation occurring in about 20 % of patients with GBM [75]. In this study, 10 patients were enrolled. The manufacturing and infusion of EGFRvIII -targeted CAR-T cells were found feasible and safe, without evidence of off-tumor toxicity or CRS. This study also demonstrated that EGFRvIII CAR-T cells were able to traffick to the regions of active GBM, target the antigen of EGFRvIII, and result in specific decrease of EGFRvIII expression. On the other hand, in situ evaluation of the TME demonstrated a significantly increased expression of inhibitory molecules and infiltration of regulatory T cells (Tregs) following CART-EGFRvIII infusion, in comparison to pre-CART-EGFRvIII infusion tumor specimens. The induction of inhibitory signals is thought to have limited the clinical efficacies of the CAR-T cells [76]. In light of this result, the group plans to design a CAR-T cell that targets wild-type EGFR together with a strategy similar to the Bispecific T cell Engagers (BiTE) to bypass or re-direct Tregs. This newly engineered CAR, designated CAR-TEAM, can produce TEAM (T-cell Engaging Antibody Molecule) that targets wild type EGFR, and like BiTE, binds both EGFR on the tumor cells at the local site and CD3 on the T cells. In addition, TEAM is anticipated to be rapidly cleared in the circulation to restrain its potential cytotoxic effects on EGFR expressing normal cells and potentially re-direct Tregs. *In vitro* study did demonstrate that TEMA bound both CAR-T cells and bystander T cells. In the animal model, the CAR-TEAM cells eliminated tumors not only aggressive EGFRvIII^+^ GBM, but also EGFRvIII^-^ GBM *in vivo* [54]. There was no evidence of CAR-TEAM induced toxicity in the skin graft model. Thus, the Marcela Maus study demonstrated that the CAR-TEAM T cell therapy can potentially overcome the heterogeneity of EGFRvIII expression in GBM while taking advantage of EGFRvIII-directed CAR-T cell trafficking into the tumors and that TEAMs secreted by CAR-T cells do not have systemic on-target toxicities. For the GBM treatment, it is expected that local, multi-dose and multi-target approaches with CAR-TEAM are likely to be more effective and lead to durable response. Nevertheless, whether this engineered CAR-EGFRvIII has a superior efficacy and safety profile will still need to be tested in the clinical study which is being planned.

### Breaking barriers for T cell-based cellular therapies in solid tumors

While Prasad Adusumilli, Marcela Maus and many groups continue to strive in making CARs more accessible to solid tumors, others including Stanley Riddell from Fred Hutchinson Cancer Research Cancer have made an indispensable, complementary advancement in breaking the barriers in the solid tumors to make them more acceptable for CAR-T cells. Similar strategies are anticipated to be applicable for T cell-based cellular therapies for solid tumors in general.

The studies on the receptor tyrosine kinase-like orphan receptor 1 (ROR1) have uncovered the fundamental differences between solid and “liquid” tumors in their acceptability to the same CAR-T cells. ROR1 is expressed during embryonic development and in some normal tissues, but overexpressed in many incurable-stage, common solid tumors and is associated with poor prognosis possibly through regulating tumor growth and metastasis [77]. ROR1 CAR-T cells were safe in non-human primates and demonstrated efficacy in pre-clinical tumor models [78]. A phase I clinical trial of ROR1 CAR-T cells was conducted in patients with refractory ROR1^+^ lung cancer, TNBC, and hematologic malignancies (NCT02706392). While two of two chronic lymphocytic leukemia (CLL) patients had clinical response with PR and CR, respectively, only 1/14 patient with solid tumors had PR (unpublished data from Stanley Riddell). In the CLL patients, CAR-T cells had marked expansion in the blood, infiltrated tumor sites (bone marrow compartment), and eradicated ROR1^+^ CLL cells in the bone marrow. The CAR-T cells had increased expression of PD-1 after the infusion, but not the other inhibitory molecules, such as Lag3, Tim3 and TIGIT. In the solid tumor patients, CAR-T cells expanded and proliferated well *in vivo*, but infiltrated tumors poorly and had upregulation of all the inhibitory receptors tested. These CAR-T cells, at the peak of the possible response window, still lacked effector function as measured by production of anti-tumor cytokines such as INFγ, TNFα, and GM-CSF and exhibited the transcriptional features of exhausted T cells. These results elicited the obstacles for T cell therapy of solid tumors, including failure of trafficking to tumors, T cell exhaustion induced by tumor-associated chemokines, in addition to other immunosuppressive cells and factors in the TME [79].

In the KRAS oncogenic mutation and p53 loss of function mutation (KP) conditional knock-in mouse model of non-small cell lung cancer (NSCLC), tumors co-evolve naturally with host’s immune system in a clinically relevant TME is used by the Riddell group to explore the strategy to overcome resistance of CAR-T cell therapy. ROR1 was introduced into the KP NSCLC cells for studying the ROR1 CAR-T cells in the treatment of ROR1^+^ NSCLC. The KP^ROR1^ tumor develops a TME similar to a cold tumor subtype of human NSCLC characterized by few lymphocyte infiltration, abundant myeloid infiltration, and development of tertiary lymphoid structures enriched with Foxp3^+^ Tregs in a low CD8/Treg ratio. When these mice were treated with ROR1 CAR-T cells, there was minimal tumor response. The possibility of ROR1 escape was excluded as the tumors cells remained strongly positive for ROR1. ROR1 CAR-T cells also expanded in the blood, but infiltrated the lung tumors poorly, and had upregulation of inhibitory receptors on the T cells resembling the situation of ROR1 CAR-T cells founded in NSCLC patients in the above clinical trial. Moreover, addition of anti-PD-1 or anti-PD-L1 antibodies did not result in improvement of tumor response.

Thus, the above mouse model becomes a preclinical platform to study whether the infiltration of CAR-T cells into tumors could be enhanced by adding conventional treatments to the lymphocyte-depletion conditioning regimen prior to infusion of CAR-T cells. Conventional treatments tested by the Riddell group included anthracyclines and platinum-based chemotherapeutic agents and radiotherapy, which are known to induce immunogenic cell death characterized by the release of calreticulin, ATP and HMGB1, activation of DCs/macrophages in the draining lymph nodes, and induction of chemokines and pro-inflammatory cytokines. These processes could result in an increased infiltration of T cells into the tumor. On the other hand, chemotherapy and radiotherapy could sensitize the tumors cells to killing by Granzyme B from the effector T cells. As an example, the combination of oxaliplatin and cyclophosphamide (Ox/Cy) was shown to induce immunogenic cell death and to improve T cell infiltration into tumor *in vivo* [53]. The Riddell group demonstrated that administration of Ox/Cy to KP^ROR1^ mice could induce expression of T cell-recruiting chemokines within the KP^ROR1^ tumors, and markedly enhanced the infiltration of ROR1 CAR-T cells into the tumors. Due to the observation of significant infiltration of myeloid suppressive cells with high expression of PD-L1, anti-PD-L1 antibody was given after the infusion of CAR-T cells. Ox/Cy and anti-PD-L1 significantly enhanced and sustained the CAR-T cell infiltration into the tumor *in vivo* [80].

Lung tumors were harvested on day 10 and day 56 after Ox/Cy and CAR-T cell infusion for single cell RNA sequencing (scRNAseq) analysis. Ox/Cy not only enhanced the infiltration of T cells into the tumor, but also caused the dynamic changes in the macrophage clusters. The dominant inhibitory alveolar macrophages population inside the tumors changed to a new cluster of macrophages with enhanced phagocytosis, antigen presentation, IFNγ and TNFα and Toll-like receptor signaling, most importantly with increased expression of chemokines for recruitment of T cells (CCL4 and CXCL16) and monocytes (CCL2 and CCL7), and with decreased expression of chemokines for neutrophil recruiting (CXCL3 and CXCL15). After infusion of CAR-T cells, the macrophages were further switched to a dominant cluster of activated macrophages in the tumors on day 10. This cluster of macrophages had IFNγ responding signatures, upregulated Nos2, as well as CXCL9 and CXCL10 chemokines which may attract a second wave of CAR-T cells into the tumor. These observations explain how Ox/Cy and anti-PD-L1 antibody may markedly improve tumor control by ROR1-targeting CAR-T cells clinically. It should be noted that the improved survival did not lead to a cure, likely due to, on day 56, the repopulation of alveolar inhibitory macrophages in the TME and their expression of inhibitory TGFβ1, IL-10, SPP1, and neutrophil-recruiting chemokines (CXCL3, CXCL15), and declination of CAR-T cells in both number and function, leading to tumor progression. Thus, additional strategies would need to be developed to sustain the macrophage shift within the TME to sustain the CAR-T cells in the long term. Understanding the mechanisms by which the inhibitory alveolar macrophages re-populate would anticipate improving the efficacy of CAR-T cells to cure the tumors. Although a tumor-specific genetic engineered mouse model was used in above studies, the same concepts are anticipated to be applicable for other solid tumors and warrant further investigation in the preclinical models of other solid tumors and in the clinical studies for CAR-T and other T-cell based cellular therapies.

## Conclusions and future prospective

Cellular therapy has shown great success in treating hematologic malignancies. Solid tumors pose unique challenges that require further engineering of T cells and manipulation of the TME to achieve a success. The biological complexity and potential crosstalk among different engineered features within the T cells, as well as among engineered and endogenous immune cells, tumor cells, and other tumor-intrinsic and extrinsic factors, must be carefully examined for broad application of cell therapy in cancer. The capacity of next-generation and single-cell sequencing technologies, as well as proteomic and metabolomic analysis techniques, had significantly enhanced our ability of understanding and rationally manipulating these complex interactions. However, preclinical findings need to be carefully and rationally tested in the clinical trial settings. Conventional therapies such as chemotherapy and radiotherapy should be considered for their roles in manipulating TME but more work needs to be done to optimize their roles in enhancing effective T cell therapies. The current clinical development should undoubtedly focus on identifying new indications for T cell therapy, developing safer T cell therapy platforms, and manufacturing T cell therapy more efficiently. While logistic challenges remain, personalized T cell therapy against individualized cancer-specific neoepitopes could provide potential cure to every individual patients of solid tumors. The growing toolbox of T-cell engineering strategies that can be synergistically deployed and modularly calibrated for maximum safety and efficacy will continue to enable innovations that aim to generate new treatment options for currently intractable diseases.

## Data Availability

The authors agree to open access for this publication.

## Abbreviations

BCMA: B cell maturation antigen
CAHON: Chinese American Hematologist and Oncologist Network
CAR-T: T cells expressing chimeric antigen receptor
ETC: Endogenous T cells
HLA: Human leukocyte antigen
ICI: Immune checkpoint inhibitor
NSCLC: Non-small cell lung cancer
PD-1: Programmed cell death protein 1
PD-L1: Ligand 1 from programmed cell death protein 1
